# Consensus on targeted drug therapy for spondyloarthritis

**DOI:** 10.2478/rir-2023-0009

**Published:** 2023-07-22

**Authors:** Xinping Tian, Mengtao Li, Shengyun Liu, Xiaomei Leng, Qian Wang, Jiuliang Zhao, Yi Liu, Yan Zhao, Yizhi Zhang, Huji Xu, Jieruo Gu, Xiaofeng Zeng

**Affiliations:** Department of Rheumatology and Clinical Immunology, Peking Union Medical College Hospital, Peking Union Medical College, Chinese Academy of Medical Sciences, National Clinical Research Center for Dermatologic and Immunologic Diseases, Ministry of Science & Technology, State Key Laboratory of Complex Severe and Rare Diseases, Key Laboratory of Rheumatology and Clinical Immunology, Ministry of Education, Beijing 100730, China; Department of Rheumatology and Immunology, the First Affiliated Hospital of Zhengzhou University, Zhengzhou 450052, Henan Province, China; Department of Rheumatology and Immunology, West China Hospital Sichuan University, Chengdu 610041, Sichuan Province, China; Department of Rheumatology and Immunology, the First Affiliated Hospital of Harbin Medical University, Haerbin 150001, Heilongjiang Province, China; Department of Rheumatology and Immunology, Shanghai Changzheng Hopital, Shanghai 200003, China; Department of Rheumatology and Immunology, the Third Affiliated Hospital of Sun Yat-sen University, Guangzhou 510630, Guangdong Province, China

**Keywords:** spondyloarthritis, targeted drug, consensus

## Abstract

Spondyloarthritis (SpA) is a group of chronic inflammatory diseases that predominantly involve the spine and/or peripheral joints. The clinical manifestations of SpA are highly heterogenous and complicated with various comorbidities. SpA is a disabling disease and adversely affects the quality of life of patients. Many new medications that target cytokines or pathways specific for the pathogenesis of SpA have been developed and they are becoming increasingly important in the treatment of SpA. However, identifying the target patient population and standardizing the usage of these drugs are critical issues in the clinical application of these “targeted therapeutic drugs”. Under the leadership of National Clinical Research Center for Dermatologic and Immunologic Diseases (NCRC-DID), managed by Peking Union Medical College Hospital, the “Consensus on targeted drug therapy for spondyloarthritis” has been developed in collaboration with the Rheumatology and Immunology Physicians Committee, Chinese Medical Doctors Association, Rheumatology and Immunology Professional Committee, Chinese Association of Rehabilitation Medicine, and Chinese Research Hospital Association Rheumatology and Immunology Professional Committee. This consensus has been developed with evidence-based methodology and has followed the international standard for consensus development.

Spondyloarthritis (SpA) is a group of chronic inflammatory diseases that predominantly involve the spine and/or peripheral joints. SpA is a disabling disease with highly heterogeneous clinical manifestations. In general, SpA encompasses two clinical subtypes: axial SpA (ax-SpA) includes ankylosing spondylitis (AS) and non-radiographic ax-SpA (nr-ax-SpA), and peripheral SpA includes psoriatic arthritis (PsA), inflammatory bowel disease-associated arthritis, reactive arthritis, undifferentiated SpA, and juvenile SpA. It has long been observed that SpA is associated with many comorbidities, such as psoriasis and inflammatory bowel disease (IBD); it is also associated with cardiovascular, cerebrovascular diseases and other major complications, which adversely affect the quality of life and physical function of patients.^[1]^ The prevalence of SpA in China is about 0.93%^[2]^ and tends to rise in recent years.

In the past two decades, the treatment of SpA has shifted from non-specific anti-inflammation to specific therapy targeting inflammatory cytokines or cellular pathways that play an important role in the pathogenesis of SpA, *i.e*., targeted therapy. Targeted therapeutic drugs can rapidly relieve clinical symptoms, delay disease progression, improve the patients’ quality of life and reduce disability. At present, the targeted drugs approved for the treatment of SpA in China and abroad mainly include biologics and synthetic small molecule targeted drugs. Biologic agents include tumor necrosis factor (TNF) -α inhibitors (etanercept, infliximab, adalimumab, golimumab, certolizumab pegol), interleukin (IL) -12/IL-23 inhibitors (ustekinumab, guselkumab), IL-17A inhibitors (secukinumab, ixekizumab) and selective T cell co-stimulation modulators (abatacept). Synthetic small molecule targeted drugs mainly include Janus kinase (JAK) inhibitors (such as tofacitinib, upadacitinib among others) and phosphodiesterase 4 inhibitors (apremilast).

At present, there is a wide variety of targeted drugs that have been marketed in China, with different mechanisms of action, approved indications and contraindications. In order to standardize the application of targeted drugs in the treatment of SpA in China, this consensus is formulated by the National Clinical Research Center for Dermatologic and Immunologic Diseases (NCRC-DID) in conjunction with the Rheumatic Immunology Physicians Branch of Chinese Medical Doctor Association, the Rheumatic Immunology Specialized Committee of Chinese Society of Rehabilitation Medicine and the Rheumatic Immunology Specialized Committee of Chinese Research Hospital Association. This is evidence-based and has followed international norms for consensus formation.

## Method

NCRC-DID has taken the initiative to develop this consensus. This consensus has been developed according to the international consensus development process. First, a panel of experts in rheumatology nationwide was formed and they picked up clinical problems that needed to be standardized in SpA targeted drug therapy, which were condensed into 13 clinical problems after three-round Delphi. The consensus was drafted based on 13 questions, following the principles of population, intervention, active comparator (measure), prognosis (population, intervention, comparator, outcomes [PICO]). Such search engines as PubMed, EMBASE, Wanfang database, and China National Knowledge Infrastructure (CNKI) were used, and all published SpA-related guidelines were referenced. Systematic reviews, expert consensus, and original studies before January 2022 were reviewed as evidence. Three expert meetings were organized on July 15, 2021, June 24, 2022, and September 7, 2022 to discuss and revise recommendations to form this version of consensus.

## Consensus

***Recommendation 1: For patients with ax-SpA who fail to respond to at least two non-steroidal anti-inflammatory drugs (NSAIDs) successively, or patients with SpA with poorly controlled peripheral joint disease after being treated with one conventional synthetic disease-modifying antirheumatic drug (csDMARDs), or patients with PsA with severe skin lesions, targeted drug therapy is recommended***

Targeted drug therapy is recommended for patients with active ax-SpA who were treated with at least two NSAIDs for more than 4 weeks at the maximum dose, or who have been treated with two NSAIDs for 2 months or intolerant to NSAIDs. Targeted drug therapy is also recommended for patients with SpA who have poorly controlled peripheral arthritis despite being treated with one csDMARD, or persistent active inflammation and axial symptoms despite being treated with one of the NSAIDs. SpA disease activity is defined as AS disease activity score based on C-reactive protein (ASDAS-CRP) ≥ 2.1 or Bath AS activity index (BASDAI) ≥ 4. Targeted drug therapy is also recommended for PsA patients with severe skin lesions.

Studies have shown that the maximum dose of NSAIDs is required to achieve the best response, and it usually takes 2 weeks to reach the maximum response as no further response was observed after 2 weeks.^[3,4]^ Guidelines released in the literature recommended a similar time frame to start targeted drug therapy in patients with AS. In 2016, the International SpA Assessment Collaboration/European Alliance of Associations for Rheumatology (EULAR) recommended that biologics should be initiated after failure to respond to at least two NSAIDs for more than 4 weeks cumulatively for patients with SpA.^[5]^ In 2019, the American College of Rheumatology (ACR) recommendations for SpA stated that biologics should be started when treatment is ineffective or intolerant after being treated with at least two NSAIDs at the maximum dose for more than 1 month, or not effective after at least two NSAIDs are used successively for more than 2 months.^[6]^

For patients with PsA who have predominantly peripheral joint involvement, targeted drug therapy can be used as a first line treatment, in particular when enthesitis or axial symptoms are prominent.^[7]^ When psoriatic skin lesions are extensive and severe, or involve specific areas such as face, scalp, nails, and genitals, biologics may also be administered as the first line therapy.^[8]^

***Recommendation 2: Before starting targeted drug therapy, it is recommended to undergo a comprehensive evaluation of disease activity, progression of structural damage, location and extent of joint involvement, extra-articular manifestations, functional status, and concomitant diseases for all patients with SpA***

Patients with SpA should be comprehensively evaluated prior to initiation of targeted drug therapy as disease activity is the key determining factor. The commonly used assessment tools for ax-SpA include the AS disease activity score (ASDAS) or BASDAI, and PsA disease activity index for PsA (DAPSA) or minimum disease activity (MDA) for PsA. Enthesitis is considered a characteristic lesion^[9, 10, 11]^ and the basis for structural progression of SpA due to its potentially irreversible bone damage.^[9]^ The hip is commonly affected in patients with ax-SpA, resulting in joint structure damage, and limited physical function and disability. The distal interphalangeal joint is most commonly affected in PsA, which can progress from mono- or oligo- arthritis to polyarthritis and eventually can lead to disability. Therefore, targeted drug therapy should be started as early as possible in SpA patients with enthesitis or hip joint involvement, and in PsA patients with distal interphalangeal arthritis.^[12]^

Extra-articular manifestations of SpA, such as uveitis and concomitant inflammatory bowel disease and psoriasis,^[13]^ are associated with different cytokine pathways. For example, TNF-α plays an important role in the pathogenesis of intestinal lesions and uveitis, whereas IL-23/IL-17 pathway plays a major role in skin lesions. Therefore, targeted therapeutic drugs may be selected based on different clinical manifestations.^[14]^

***Recommendation 3: Chronic hepatitis virus infection and tuberculosis infection should be screened prior to initiation of targeted drug therapy***

TNF-α is an important cytokine involved in inhibiting HBV viral replication. Approximately 5% of patients with AS or PsA with a history of HBV and inflammatory bowel disease, receiving TNF inhibitors were reported to have HBV reactivation, liver failure, or even death in severe cases. The risk of hepatitis virus reactivation in HBsAg-positive patients can be as high as 27% to 39%.^[15]^ JAK inhibitors also increase the risk of HBV viral replication. There has been no report on HBV reactivation in several clinical trials of IL-17A inhibitors in SpA patients.^[16, 17, 18, 19, 20]^ Patients with chronic HCV infection have a relatively low risk of viral reactivation when being treated with targeted drug therapy. However, the long-term safety of IL-17A inhibitor use is not clear. Therefore, it is recommended that all SpA patients should be screened routinely for HBV and HCV infection before starting targeted drug therapy.

Latent tuberculosis infection in Chinese patients with SpA has been reported to be as high as 25%.^[21]^ A meta-analysis has shown that patients with inflammatory arthritis treated with TNF inhibitors have a 3-fold increased risk of reactivation of tuberculosis infection compared with controls.^[22]^ JAK inhibitors have a similar risk to TNF inhibitors. Screening for tuberculosis infection is, therefore, recommended before initiating targeted drug therapy. Several large long-term follow-up studies of IL-17A inhibitors for SpA did not find reactivation of tuberculosis infection.^[17,23, 24, 25, 26, 27, 28]^

***Recommendation 4: It is recommended to start TNF inhibitors or IL-17A inhibitors as the first line therapy for patients with ax-SpA. Different targeted drugs may be chosen for patients with PsA based on clinical manifestations***

Several randomized controlled trials conducted in patients with AS have shown that TNF inhibitors can effectively improve the clinical manifestations and imaging findings of patients with active AS, in view of composite efficacy indicators, patient-reported outcomes and imaging changes. IL-17A inhibitors can also effectively control the inflammation of AS and improve clinical symptoms and imaging changes. At present, there are no head-to-head comparative studies available on TNF inhibitors versus IL-17A inhibitors for the treatment of AS, but guidelines from both China and abroad recommended that TNF inhibitors or IL-17A inhibitors can be used without preference of one over another after NSAIDs failure.^[29,30]^ TNF inhibitors and IL-17A inhibitors have been approved by the US Food and Drug Administration for the treatment of nr-ax-SpA, but not yet in China.

Peripheral arthritis, enthesitis, dactylitis, skin lesions, and nail lesions are common in patients with PsA, and targeted drugs should be selected according to the sites of involvement and severity. At present, several phase III clinical trials in PsA have shown that TNF inhibitors,^[31, 32, 33, 34, 35, 36, 37, 38, 39, 40]^ IL-17A inhibitors,^[41, 42, 43]^ IL-12/ IL-23 inhibitors, JAK inhibitors, phosphodiesterase 4 (PDE4) inhibitors, and selective T cell co-stimulation modulators can effectively improve peripheral arthritis^[44, 45, 46, 47, 48, 49, 50, 51, 52]^ and psoriatic lesion.^[31, 32, 33, 34, 35, 36, 37, 38, 39, 40,51,53, 54, 55]^ Two head-to-head studies of IL-17A inhibitors versus TNF inhibitors in patients with PsA have shown that IL-17A inhibitors are superior to TNF inhibitors in improving skin lesions but with similar in joint disease improvement.^[54,56]^ Therefore, IL-17A inhibitors are recommended preferentially when skin lesions are the predominent clinical features. Approximately 80% of patients with PsA have nail involvement.^[57]^ Limited phase III clinical trials have shown that TNF inhibitors^[32,34]^ or IL-17A inhibitors^[58, 59, 60, 61]^ could improve nail damage in patients with PsA. IL-17A inhibitors are effective in the treatment of enthesitis. The results of two phase III clinical trials,^[42]^ in which 477 patients with PsA were recruited and treated with secukinumab for 16 weeks, have shown that 65% (300 mg secukinumab) and 56% (150 mg secukinumab) of patients achieved complete resolution of enthesitis, respectively, and the efficacy lasted for 2 years, with a shorter time to remission than the placebo.^[62]^ In addition, phase III clinical trials have shown that secukinumab or ixekizumab could improve the dactylitis as well.^[41,55]^ TNF inhibitors,^[32,34,39,63]^ ustekinumab and guselkumab,^[44,47,64]^ are effective due to their inhibitory effects on IL-23 in the treatment of enthesitis and dactylitis.

Abatacept inhibits T cell activation by blocking the costimulatory signals, which in turn inhibits B cell proliferation and differentiation, and reduces the production of autoantibodies.^[52,65]^ In a phase II/III clinical trial of abatacept in the treatment of PsA, a higher proportion of adult patients with active PsA previously treated with DMARDs and TNF inhibitors achieved the American College of Rheumatology (ACR) -defined 20% remission (ACR20) at 24 weeks compared with placebo.^[52,65]^ Abatacept is an alternative treatment for patients with PsA, who have a history of serious infection or recurrent infection, but it is preferred to be administered after IL-17A inhibitors or IL-12/IL-23 inhibitors. Abatacept may be used for patients with PsA who respond poorly or intolerant to TNF inhibitors, are complicated with congestive heart failure, or demyelinating disease.^[66]^

PDE4 inhibitors have also been shown to improve enthesitis and dactylitis in patients with PsA. Clinical trials in patients with PsA have shown significant symptomatic improvement at the dose up to 30 mg/day.^[67]^

In clinical trials, JAK inhibitors have been shown to have good efficacy in patients with PsA. Tofacitinib and upadacitinib are currently available with evidence of efficacy and safety in the treatment of PsA. In a 6-month randomized, double-blind, placebo-controlled, phase III trial,^[68]^ compared with placebo, higher proportion of patients with active PsA who did not respond adequately to TNF inhibitors achieved ACR20 response when being treated with tofacitinib for 6 months. In another 12-month, phase III, randomized, controlled trial,^[69]^ tofacitinib and adalimumab were compared in patients with PsA, who did not respond adequately to more than one conventional DMARDs; this study has shown that the proportion of patients achieving ACR20 in the tofacitinib treatment group was not significantly different from adalimumab treatment group.

In addition to tofacitinib, upadacitinib, which selectively acts on JAK1 kinase, also has good efficacy for PsA. A multicenter, randomized, controlled (adalimumab as the control) trial in patients with PsA, who had inadequate response to more than one non-biologic DMARDs, has shown that upadacitinib is effective in improving manifestation of PsA^[70]^ at 56 weeks of treatment. The proportion of patients achieving responses were similar in both upadacitinib and adalimumab arms based on ACR20, ACR 50% response to PsA (ACR50), ACR 70% response to PsA (ACR70), psoriasis area and severity index (PASI) 75% response (PASI75), PASI 90% response (PASI90), and PASI 100% response (PASI100). In another multicenter, randomized, double-blind, placebo-controlled, phase III clinical trial,^[71]^ significant symptomatic improvement was observed in patients with PsA, who did not respond well or were intolerant to biologics after treatment with upadacitinib for 2 weeks. The proportions of patients achieving ACR20, ACR50, ACR70, PASI75, PASI90, PASI100 and other indicators were similar in both groups at 56 weeks of treatment. No new safety risk signals were identified. Upadacitinib has been approved for the treatment of PsA in China.

***Recommendation 5: It is recommended to prefer monoclonal antibody TNF inhibitors for SpA patients complicated with recurrent uveitis or inflammatory bowel disease over fusion protein TNF inhibitors***

AS, nr-ax-SpA and PsA patients with anterior uveitis are 23%,15.9%, and 3% AS, nr-ax-SpA and PsA patients with anterior uveitis respectively,while 6.4%, 4.1%, and 3% of those patients with inflammatory bowel disease; and 10.2% and 10.9% in AS and nr-ax-SpA patients with psoriasis.^[72]^ Clinical studies and meta-analysis of the treatment of SpA patients showed that adalimumab and infliximab could reduce the occurrence of uveitis in AS patients compared with etanercept. A small number of open-label and uncontrolled studies in patients with SpA have shown efficacy of certolizumab pegol or golimumab in the treatment of SpA-associated anterior uveitis. These data indicate that TNF-α monoclonal antibodies are superior to the TNF-α fusion protein etanercept in preventing recurrent uveitis. The role of IL-17A inhibitors in the prevention and reduction of anterior uveitis recurrence is not clear. There are reports of inflammatory bowel disease induced or aggravated by IL-17A inhibitors, thus IL-17A inhibitors should be used with caution in SpA patients with active inflammatory bowel disease.

***Recommendation 6: JAK inhibitors may be an option for patients who have had inadequate response or intolerant to TNF inhibitors or IL-17A inhibitors***

JAK inhibitors have been shown to have good efficacy in clinical trials in patients with AS by inhibiting signaling transduction through intracellular JAK pathway. JAK inhibitors can relieve enthesitis and dactylitis. Currently available JAK inhibitors with evidence of efficacy and safety in the treatment of SpA are tofacitinib and upadacitinib, which can be used as an option for patients who fail or have suboptimal efficacy or intolerance to other targeted drugs for SpA.^[73, 74, 75, 76]^ A multicenter, randomized, double-blind, placebo-controlled phase III clinical trial has shown that in adult patients with active AS, tofacitinib has significant clinical efficacy noticeable from the second week; tofacitinib treatment group achieved 20% AS remission (ASAS20) as established by the Assessment of Spondyloarthritis International Society (ASAS) and 40% (ASAS40) after 16 weeks of treatment. These remission scores were significantly higher than those in the placebo group. Tofacitinib has recently been approved by the National Medical Products Administration for the treatment of adult patients with active AS who have inadequate response or intolerance to one or more TNF inhibitors.

In a multicenter, randomized, double-blind, parallel, placebo-controlled, phase II/III trial,^[74]^ adult patients with active AS, who were biologic naive and had an inadequate response to at least two NSAIDs or intolerance/contraindication to NSAIDs, were treated with upadacitinib or placebo.^[74]^ After treatment for 14 weeks, the proportion of patients achieving ASAS40 in the upadacitinib group was twice as high as that in placebo group. After 64 weeks, the proportion of patients achieving ASAS40 could reach 85%. In another randomized, double-blind, placebo-controlled phase III clinical study, patients with nr-ax-SpA^[76]^ received upadacitinib and had a significantly higher proportion achieving ASAS40 at week 14 than those with placebo. Upadacitinib significantly improved symptoms and physical function, with better improvement in spinal scores as developed by the Spondyloarthritis Research Consortium of Canada (SPARCC) for magnetic resonance imaging (MRI) than placebo.

***Recommendation 7: It is not recommended to combine biologics with methotrexate for the treatment of ax-SpA***

In patients with SpA, previous studies have shown that methotrexate, sulfasalazine, or leflunomide is ineffective in axial symptoms and is only used for peripheral joint involvement and extra-articular manifestations if no other drug options are available due to side effects or contraindications. In patients with ax-SpA, concurrent use of these DMARDs with biologic did not result in improved outcomes even on a prolonged drug use.^[46,47]^ In randomized controlled trials in patients with ax-SpA, compared with infliximab monotherapy, infliximab combined with methotrexate did not increase the proportion of ax-SpA patients achieving ASAS20, ASAS40, or BASDAI improvement.^[77]^ In addition, pharmacokinetic studies have shown that infliximab plasma concentrations are not raised in ax-SpA patients treated with a combined therapy of infliximab and methotrexate.^[78]^

***Recommendation 8: Assessment of therapeutic response and monitoring of adverse reactions regularly is recommended during treatment with targeted drugs***

The primary goals of treating patients with SpA are to maximize health-related quality of life, preserve/normalize physical function and social participation, reduce disability, and avoid potential side effects of drugs by controlling inflammation, prevent and delay structural damage. During targeted drug therapy, it is recommended that patients who do not achieve treatment goals should be assessed every 1-3 months until treatment goals are achieved. For patients who have achieved treatment goals, disease activity should be assessed every 3-6 months. ASDAS has a good correlation with clinical efficacy, inflammatory biomarkers, and inflammation scores based on MRI and can be used to assess the efficacy of targeted drugs. Thus ASDAS or BASDAI is recommended as a tool to assess and monitor disease activity in patients with ax-SpA. In addition, patients’ overall functional status and extra-articular manifestations should be assessed regularly during treatment. Assessment and monitoring of disease activity in patients with PsA is recommended using the disease activity index for PsA (DAPSA) or the minimal disease activity (MDA).

Drug-related adverse reactions should be closely monitored. TNF inhibitors can cause upper respiratory tract infection, cytopenia, and elevated liver enzymes. Congestive heart failure and demyelinating diseases are rare. IL-17A inhibitors can cause upper respiratory tract infection, nasopharyngitis, headache, Candida infection, and inflammatory bowel disease have also been reported in some patients. JAK inhibitors also increase the risk of upper respiratory tract infection, and the rate of herpes zoster virus infection is higher than TNF inhibitors. Patients with risk factors for cardiovascular and cerebrovascular diseases have an increased risk of major cardiovascular events and thrombosis, therefore these risk factors should be taken into consideration when using JAK inhibitors.

***Recommendation 9: Targeted therapy drug taper should only be considered in patients with stable SpA on targeted drugs for at least 6 months. The recommended tapering regimen is to decrease dosages or prolong dosing interval. SpA disease activity should be assessed regularly during tapering and the last effective dosage should be restored if the disease is relapsed***

The SpA International Task Force in 2017 stated that clinical remission of musculoskeletal symptom and extra-articular manifestations (*i.e*., inactive disease) should be the treatment target of SpA. Low/very low disease activity was also an acceptable alternative treatment target.^[79]^ ASDAS-CRP < 1.3 or BASDAI ≤ 2 after treatment are considered to be the target for disease remission, and ASDAS-CRP < 2.1 or BASDAI < 4 as low disease activity for patients with ax-SpA.^[30]^ Although the current concept of treat-to-target for SpA remains controversial, tapering of targeted drugs in SpA patients may be attempted after low disease activity or disease remission has been achieved. There are few studies on tapering or discontinuing targeted drugs in SpA patients. However, studies in patients with PsA and ax-SpA showed a positive correlation between low disease activity before tapering and the likelihood that remission could still be maintained after tapering.^[80]^ Therefore, it is recommended that patients with SpA may be considered for targeted drug taper until low disease activity or disease remission is maintained for more than half a year. In addition to disease activity, the degree of extra-articular manifestations remission in patients with SpA should be considered to decide about tapering. Tapering regimens include decrease dosages or increase dosing interval.

***Recommendation 10: It is recommended to apply individualized and stratified management strategy when determining the timing and selecting targeted drugs***

Because the clinical presentations of SpA are very heterogenous, it is recommended to apply the stratified and individualized management principle when determining the timing and selecting targeted drugs.

*For SpA patients with active tuberculosis infection, targeted drug therapy is not recommended*.

For Tuberculosis (TB), it is recommended that patient should be referred to specialists for anti-tuberculosis treatment. SpA patients with latent and previous history of tuberculosis infection should receive prophylactic anti-tuberculosis therapy before initiation of targeted drug therapy. IL-17A inhibitors are preferred.

Several studies have shown an increased risk of tuberculosis infection in SpA patients treated with infliximab, adalimumab, or etanercept, with 3- to 4- fold increase of tuberculosis recurrence for patients treated with infliximab or adalimumab over etanercept.^[81]^ The Expert Group on Tuberculosis Prevention and Management in China recommended that patients with latent tuberculosis infection and previous history of tuberculosis infection should receive prophylactic anti-tuberculosis treatment for at least 4 weeks before starting TNF inhibitors. Tuberculosis infection should be re-evaluated at 3 and 6 months after treatment, and then every 6 months or 3 months after withdrawal of anti-tuberculosis drug. Patients with active tuberculosis infection should receive standard anti-tuberculosis treatment first, which should be prescribed and managed by tuberculosis specialists.^[82]^ Several large long-term follow-up clinical trials of secukinumab or ixekizumab in patients with psoriasis showed no tuberculosis recurrence.^[23,25]^ In a clinical trial in China, no active tuberculosis infection was observed in psoriatic patients with latent tuberculosis treated with secukinumab without prophylactic anti-tuberculosis therapy.^[83]^ Therefore, it is recommended that IL-17A inhibitors are preferred in patients at a high risk of tuberculosis recurrence.

*SpA patients with HBV infection should be managed based on the stratification of virus infection status*

Patients infected with HBV are at increased risk for viral reactivation throughout their entire life. Immunosuppressants and biologics are common triggers for HBV reactivation. Therefore, patients with SpA should be screened for HBV infection before initiation of biologics and be managed on a stratified approach according to HBV surface antigen (HBsAg) status. Guidelines for Prevention and Treatment of Chronic Hepatitis B (2019 Edition) in China recommend that patients with positive HBsAg should receive antiviral therapy for 1 week before starting targeted drug therapy or at least at the time of initiation of targeted therapy.^[84]^ For patients with negative HBsAg but positive anti-HBV core antibody, prophylactic antiviral therapy is needed if the HBV DNA quantification is positive. For patients with negative HBsAg, positive anti-HBV core antibody but negative for HBV DNA quantification, blood transaminases, HBV DNA quantification and HBsAg should be monitored every 1 to 3 months. Prophylactic antiviral therapy should be started immediately once HBV DNA quantification or HBsAg becomes positive. During targeted drug therapy, blood transaminases, HBV DNA quantification, and HBsAg should be monitored every 1 to 3 months.^[84]^

*For SpA patients with congestive heart failure, IL-17A inhibitors are preferred*

Etanercept tends to exacerbate pre-existing congestive heart failure. Infliximab has also been shown to significantly increase hospitalization and mortality in patients with congestive heart failure.^[85]^ IL-17A inhibitors may stabilize coronary atheromatous plaques in patients with coronary heart disease by controlling inflammation.^[86, 87, 88]^ It is recommended to prefer IL-17A inhibitors over TNF inhibitors for SpA patients with congestive heart failure.

*For SpA patients with severe active infection requiring intravenous antibiotics or hospitalization, it is recommended against continuation of targeted drug therapy*

Because targeted drugs inhibit cytokines or immune pathways in containing infectious agents, they can decrease host immune function and increase the risk of infection. A metaanalysis has shown that ax-SpA patients treated with biologics have a significantly increased risk of infection, therefore, targeted therapy is not recommended for SpA patients with severe active infection.^[89]^ Patients should be adequately assessed for risk of infection before starting targeted drugs. In case of serious infection during targeted drug therapy, biologic must be discontinued until infection is completely controlled.

*SpA patients with a history of malignancy should be thoroughly assessed for risk of cancer recurrence and targeted drugs should be used with caution*

There is no evidence that targeted drug therapy increases the risk of developing malignancy. Clinical trials of adalimumab, infliximab, golimumab, ustekinumab, or secukinumab have shown that few patients develop malignancies. Because TNF inhibitors are involved in the pathophysiology of regulating tumor growth, this effect should be taken into consideration when formulating treatment regimens for SpA patients with a history of malignancy. Several retrospective studies and meta-analyses of patients with rheumatoid arthritis treated with TNF inhibitors have shown that TNF inhibitors are not directly associated with the development of solid tumors, but the risk of developing non-melanoma skin cancer can be increased to 1.4- to 2- fold. Several clinical studies in SpA patients have suggested that IL-17A inhibitors, IL-12/IL-23 inhibitors, or JAK inhibitors do not significantly increase the risk of developing solid tumors.^[90]^ Because of controversy of targeted drug therapy in relation to the risk of developing malignancy, it is recommended that SpA patients with a history of malignancy should be thoroughly evaluated for the type, stage, risk of metastasis of previous malignancy and patient wishes before starting targeted drug therapy. Decisions should be made after carefully weighing potential benefits and risks.

***Recommendation 11: Patients with SpA may receive inactivated vaccines before or during treatment with targeted drug therapy***

Due to disease itself and drug impact, SpA patients have a higher risk of acquiring infection such as influenza and pneumonia than healthy people. For most patients with rheumatic disease on immunosuppressive therapy, vaccination is beneficial.^[91, 92, 93]^ Current clinical studies of targeted drugs in patients with rheumatic disease have demonstrated that influenza and inactivated pneumococcal vaccines are safe and effective during treatment (except for abatacept and rituximab). Therefore, pneumococcal and influenza vaccines are recommended for SpA patients before initiation of targeted drug therapy or during therapy. As there is insufficient evidence of safety with attenuated live vaccines, they are not recommended for patients with SpA.

Both TNF inhibitors and JAK inhibitors can significantly increase herpes zoster virus reactivation, with 3 to 4 cases per 100 patient-year in JAK inhibitor users and 2 to 3 cases per 100 patient-year in TNF inhibitor users. Thus, vaccination against herpes zoster is recommended 4 weeks before starting TNF inhibitors or JAK inhibitors but not during treatment. If herpes zoster develops during treatment with TNF inhibitors or JAK inhibitors, targeted therapy should be paused until viral infection is cleared. Prophylactic antiviral therapy may be considered in SpA patients with recurrent herpes zoster.

***Recommendation 12: For patients with SpA, for elective surgery, targeted therapy should be discontinued prior to operation. Therapy can be resumed at least 14 days after surgery without infection***

TNF-α plays a critical role in recruitment of inflammatory cells required for tissue repair, and treatment with TNF inhibitors may result in an increased risk of infection, impaired or delayed wound healing,^[94]^ thus preoperative biologic discontinuation is recommended to minimize the risk of surgery related infection. It is recommended that surgery should be performed after biologic discontinuation for a regular dosing interval. As there is no data available for perioperative use of IL-17A inhibitors, ustekinumab and guselkumab, preoperative discontinuation of these medications are recommended due to concern for infection and poor wound healing.^[94]^ It is recommended to discontinue tofacitinib for 3 days before surgery. For SpA patients with a previous history of prosthetic joint infection related to tofacitinib use, JAK inhibitor use is not recommended peri-operatively.^[95]^

Patients may resume targeted drug therapy if surgical wound shows signs of healing (typically around 14 days after operation) free of significant edema, erythema, exudates or infection after sutures/staples are removed.^[95]^ Recommendations for perioperative use of targeted therapy are presented in Table 1.

**Table 1:** Recommendations for perioperative use of targeted therapy in patients with SpA

Drugs	Dosing interval	Recommended timing for elective surgery (since the last dose administered)
Infliximab	Every 4, 6, or 8 weeks	Week 5, 7, or 9
Abatacept	Monthly (intravenous injection), or weekly (subcu- taneous injection)	Week 5 (intravenous injection), or week 2 (subcutaneous injection)
Certolizumab pegol	Every 2 or 4 weeks	Week 3, or 5
Rituximab	2 doses 2 weeks apart every 4~6 months	Month 5, or 7
Tocilizumab	Every week (subcutaneous injection), or every 4 weeks (intravenous injection)	Week 2 (subcutaneous injection), or week 5 (intravenous injection)
Secukinumab	Every 4 weeks	Week 5
Ustekinumab	Every 12 weeks	Week 13
Ixekizumab	Every 4 weeks	Week 5
Guselkumab	Every 8 weeks	Week 9
Tofacitinib	Daily, or twice daily	Day 4
Upadacitinib	Daily	Day 4

***Recommendation 13: Biologics that do not contain immunoglobulin (Ig) Fc fragment are preferred in pregnant and lactating female patients with SpA. Male patients may continue TNF inhibitors even for conception***

Biologics containing immunoglobulin (Ig) Fc fragment can bind to Fc segment receptors expressed in the placenta, as a result drugs can be transported from the placenta to fetal circulation leading to negative impact on fetus. However, certolizumab pegol without Fc fragment can not across the placenta and is safe to use during the entire pregnancy.^[96]^ Based on the biologic molecular structure and half-life and capability to transfer the placenta, discontinuation of infliximab and adalimumab should be recommended before 20 weeks of gestation and etanercept should be discontinued at 30-32 weeks of gestation, golimumab should be discontinued at pregnancy due to the lack of evidence of fetal safety. Studies of rheumatoid arthritis patients using a small sample size have shown that the biologic concentrations in breast milk are very low or even undetectable in patients, thus TNF inhibitors may still be considered in patients during lactation. Other targeted drugs, such as IL-17A, IL-12/IL-23 inhibitors and JAK inhibitors are not recommended in pregnant and lactating patients because there is insufficient evidence for safety during pregnancy and lactation (Table 2).^[27,97,98]^

**Table 2:** Selection of targeted drugs during pregnancy and lactation in patients with SpA

Drugs	Can be used peri-conception (Yes /No)	Can be used in the first trimester (Yes/No)	Can be used in the second/ third trimester (Yes/No)	Can be used in breastfeeding (Yes/No)
Infliximab	Yes	Yes	Stop at gestation week 20	Yes, but lack of evidence
Etanercept	Yes	Yes	can be used in first and second trimester, but should be discontinued in the third trimester	Yes, but lack of evidence
Adalimumab	Yes	Yes	Can be used in the first and second trimester, but should be discontinued in the third trimester	Yes, but lack of evidence
Certolizumab pegol	Yes	Yes	Yes, but lack of evidence	Yes, but lack of evidence
Golimumab	No data	No data	No data	No data
Ustekinumab	Yes, but lack of evidence	No data	No data	No data
Secukinumab	Yes, but lack of evidence	Yes, but lack of evidence	No data	No data

Based on the current data, men with SpA who plan for conception may continue to take TNF inhibitors (infliximab, etanercept, adalimumab and golimumab). Because there are limited data on the safety of other targeted drugs on male reproduction, targeted drugs other than TNF inhibitors are not recommended for male SpA patients for conception.

Targeted drugs are widely used in SpA patients, and it is necessary to standardize the drug use in China. This consensus has formulated the principles by which targeted drug therapy should be properly selected and used for SpA. These include selection of patient population for use, pre-medication screening, timing for initiation of therapy, drug selection and transfer, concomitant medication usage, and monitoring of adverse reactions. The consensus also emphasizes taking precautions for drug use in special patient populations. The goals are to provide guidance for clinicians to properly and safely use the targeted drugs and to improve/ standardize treatment for SpA. However, research data on targeted drug therapy for SpA is limited in China. We hope that further studies in this regard will be conducted to generate more evidence-based data before our current consensus is revised in the future.
